# Reinterpreting words and making connections: an experience with an educational book about childhood cancer

**DOI:** 10.1590/0034-7167-2024-0571

**Published:** 2026-07-10

**Authors:** Simone Lehwess Mozzilli, Danton Matheus de Souza, Milena Santos de Oliveira, Marina de Góes Salvetti

**Affiliations:** I Universidade de São Paulo. São Paulo, São Paulo, Brazil; II Instituto Beabá. São Paulo, São Paulo, Brazil; III Universidade Federal de São Paulo. São Paulo, São Paulo, Brazil

**Keywords:** Health Communication, Information Technology, Neoplasms, Pediatric Nursing, Family Nursing., Comunicación en Salud, Tecnología de la Información, Neoplasias, Enfermería Pediátrica, Enfermería de la Familia.

## Abstract

**Objectives:**

to understand the experience of children/adolescents undergoing cancer treatment, their families and healthcare professionals with the educational book “*Guia Beaba do Câncer*”.

**Methods:**

this is an exploratory, qualitative study, based on Symbolic Interactionism. Data collection was conducted online with nine children/adolescents with cancer, nine family members, and nine healthcare professionals. Semi-structured interviews were conducted, recorded, and transcribed, and analyzed using inductive thematic analysis.

**Results:**

two categories emerged: 1) Reinterpreting words: enhancing children/adolescents’ understanding of cancer with the educational book; and 2) “A watershed moment”: the educational book as a way of connecting family members to the world of cancer and professionals with children.

**Final Considerations:**

the educational book was recognized for its playful and accessible language, promoting the redefinition of experiences and symbols related to the experience of cancer, in addition to demystifying information, reducing negative reactions, and disseminating knowledge.

## RESUMEN

**Objetivos:**

comprender las experiencias de niños/adolescentes en tratamiento oncológico, sus familias y profesionales sanitarios con el libro educativo “*Guia Beaba do Câncer*”.

**Métodos:**

estudio exploratorio cualitativo basado en el Interaccionismo Simbólico. La recopilación de datos se realizó en línea con nueve niños/adolescentes con cáncer, nueve familiares y nueve profesionales sanitarios. Se realizaron entrevistas semiestructuradas, que se grabaron, transcribieron y analizaron mediante análisis temático inductivo.

**Resultados:**

emergieron dos categorías: 1) Reinterpretando palabras: mejorando la comprensión de los niños/adolescentes sobre el cáncer con el libro educativo; y 2) “Un momento decisivo”: el libro educativo como forma de conectar a los familiares con el mundo del cáncer y a los profesionales con los niños/adolescentes.

**Consideraciones Finales:**

el libro educativo fue reconocido por su lenguaje lúdico y accesible, promoviendo la resignificación de vivencias y símbolos relacionados con el cáncer, además de desmitificar información, reducir reacciones negativas y difundir conocimiento.

## INTRODUCTION

In recent years, there has been an epidemiological transition among children/adolescents, with an increase in chronic non-communicable diseases^(1)^. Childhood cancer is a notable example, having become a global public health problem. The World Health Organization estimates that more than 400,000 children between the ages of 0 and 19 are diagnosed with cancer each year^(2)^. In Brazil, it is estimated that more than seven thousand children will be diagnosed with cancer each year from 2023 to 2025, which equates to a risk of 134.8 per million children/adolescents^(3)^. Children/adolescents affected by cancer have an approximately 80% chance of being cured if diagnosed early and treated at specialized centers^(1)^. However, more than 50% of diagnoses occur at advanced stages, primarily due to a lack of awareness in the general public about the clinical presentation^(4)^.

Previous studies have reported numerous impacts of the diagnosis and course of cancer on children, adolescents, their families and social groups in which they are inserted^(5-7)^. This experience involves personal, relational, and environmental changes. It involves a disruption of future expectations and a fear of death, as well as an interruption of dreams, desires, ambitions, and achievements. Emotional reactions may include anxiety, depression, and feelings of powerlessness^(5-8)^. Several studies show that lack of information is related to these negative outcomes^(5-9)^. In this context, it is necessary to pay close attention to childhood cancer and to disseminate clear and optimistic information about this condition.

A cross-sectional study of 100 family members of children with cancer in Indonesia revealed a lack of information and gaps in support related to the disease and its treatment. The study emphasized clinical information, which hinders understanding and increases emotional reactions^(4)^. In Brazil, qualitative research with 27 family members of children with cancer demonstrated the desire for accessible and understandable information, which is a form of relief and empowerment in child care^(5)^.

In order to disseminate clear and accessible information, it is important for healthcare professionals to use soft communication technologies^(10)^. One possibility is the use of educational books, the focus of this study. These books allow for an explanation of the diagnosis, procedures, exams, and adopted therapy. This approach favors healthy coping and satisfaction among children, adolescents, and families with regard to care^(11)^.

The educational book “*Guia Beaba do Câncer*” was developed to provide clear and accessible information, demystify childhood cancer, and improve the quality of health communication. The creation of a guide named “*Guia Beaba do Câncer*” involved the active participation of children/adolescents undergoing cancer treatment, their families, healthcare professionals, and information and communication technology specialists. In 2024, the content of the educational book was successfully validated from the perspectives of children, adolescents, families, and healthcare professionals^(12)^.

The production of this educational book aligns with the emerging research priorities in the field of child/adolescent cancer this decade, highlighting studies that: understand the use of interventions for self-management of clinical conditions; optimize communication and information sharing in care; assess different support programs after the diagnosis of chronic diseases; and develop strategies to assist families in decision-making throughout the care of children/adolescents^(13)^. These priorities interconnect with the aforementioned aspects, serving as the basis for this study, which is based on the following concerns: how do children, adolescents, and families understand the use of an educational book about childhood cancer in their experience with the disease? How do healthcare professionals use the educational book?

To answer the research question, this study used Herbert Blumer’s Symbolic Interactionism theoretical framework^(14)^. This framework indicates that to understand a phenomenon in an individual’s life, one must integrate their discourse with their social interactions. In this study, for instance, children, adolescents, families, and healthcare professionals interact with each other, with themselves, with their social context, with cancer, and with the educational book. All of these factors influence their experiences.

## OBJECTIVES

To understand the experience of children/adolescents undergoing cancer treatment, their families and healthcare professionals with the educational book “*Guia Beaba do Câncer*”.

## METHODS

### Ethical aspects

This study is part of a larger project and the data comes from a master’s dissertation entitled “*Validação de livro educativo para famílias com crianças ou adolescentes em tratamento oncológico e análise da experiência*”^(15)^. The study was approved by the Research Ethics Committee of the *Universidade de São Paulo* School of Nursing. Informed Consent Form and Assent Form were obtained from all online participants. National and international ethical guidelines were respected. The discourses were identified by the letters P (patient-child and/or adolescent), F (family member) or HP (healthcare professional), followed by a number based on the order of inclusion in the study: P1, F1, HP1...

### Theoretical framework

To understand the experiences of children, adolescents, family members, and healthcare professionals, we analyzed the data using Herbert Blumer’s Symbolic Interactionism framework^(14)^. This theoretical framework assumes that perceptions are generated through interactions within the social dynamics in which individuals are immersed. These interactions are influenced by the broader context in which they occur and trigger actions that are interpreted, thereby shaping individuals’ perceptions and understanding of a specific phenomenon. In this study, the phenomenon is the experience of using the “*Guia Beaba do Câncer*”. Symbolic Interactionism has three premises that have been used: 1) Individuals’ actions are the result of the meanings that a phenomenon has for itself; 2) The meaning of the phenomenon emerges from individuals’ interactions in its collective environment; and 3) The meanings of a phenomenon can be modified from new interactions within its social dynamics.

### Study design

This is an exploratory descriptive study with a qualitative approach. The study used the COnsolidated criteria for REporting Qualitative research for its writing^(16)^.

### Study setting and recruitment

The study was conducted remotely, via the Zoom^®^ platform, between December 2020 and January 2021. The online approach was chosen to include children, adolescents, family members, and healthcare professionals from different locations in Brazil who had come into contact with the educational book. This approach was also chosen because of the declaration of the pandemic, which reduced the number of people circulating in the researchers’ oncology hospital.

The study population consisted of children/adolescents who had experienced cancer during childhood or adolescence, as well as their family members and healthcare professionals. The study included children/adolescents aged four years or older who were undergoing oncological treatment, in the maintenance phase, or in partial or complete remission of the disease. Family members aged 18 or older who were immediate family members of the child and self-identified as literate were included. As for healthcare professionals, those with at least 24 months of experience directly caring for children/adolescents with cancer were included. All participants should have had contact with and/or used the educational book “*Guia Beaba do Câncer*” and have internet access. Individuals with communication difficulties, cognitive impairment, or disabling diseases were excluded from the study.

During the study period, the second edition of the “*Guia Beaba do Câncer*” was available. The first edition included 2,250 copies, which were distributed directly to oncology hospitals throughout Brazil. The second edition included 4,250 copies, which were distributed directly to hospitals, as well as to 163 patients, families, and professionals who requested the guide via email from the “*Beaba*” institution and received it by mail. Their email addresses were registered. For the main study mentioned above, invitations to participate in the guide validity study were sent by email to these 163 registered email addresses^(12)^.

To ensure continuity of the research process through qualitative data collection with individuals who were already familiar with the research topic, we chose to invite participants who had already participated in the previous content validity stage. Thus, we invited 12 children/adolescents, 20 family members, and 29 healthcare professionals who had participated in the previous validity study to participate via email^(12)^. However, only nine children/adolescents, nine family members, and nine healthcare professionals who used the educational book “*Guia Beaba do Câncer*” during cancer treatment responded to the invitation and agreed to participate, constituting the study’s sample.

### Data collection and organization

**Figure 1:**
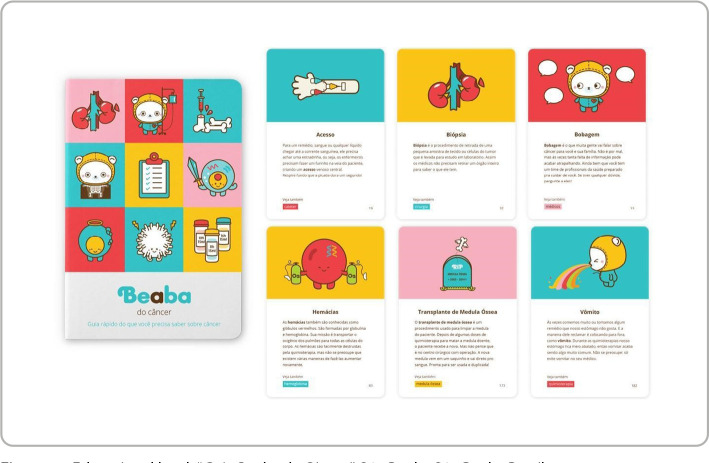
Educational book “*Guia Beaba do Câncer*”, São Paulo, São Paulo, Brazil, 2022

Initially, researchers created and validated the educational book “*Guia Beaba do Câncer*”^(12)^. The book is part of the educational materials of the Public Interest Civil Society Organization, *Beaba*, which operates throughout Brazil and is composed of patients, family members, and healthcare professionals. The organization works to disseminate complex information in clear, accessible, and optimistic language. The book is structured like a dictionary of oncological terms and is organized alphabetically. Words such as “red blood cells”, “neutropenia”, and “cerebrospinal fluid” are explained clearly and humorously. Figure 1 exemplifies the book’s illustrations.

The educational book is promoted on the organization’s social media channels and is available to children, adolescents, and their families free of charge after a donation is requested on the organization’s website. Print copies are also available at healthcare institutions affiliated with the organization.

Semi-structured interviews were conducted for data collection. These interviews were pre-scheduled and recorded using the Zoom^®^ platform. The interviews were conducted individually with children or adolescents, their families, and healthcare professionals. Two female researchers, both psychology graduates with experience conducting qualitative studies and previously trained by the principal researcher, conducted the interviews. One of the researchers was part of the Beaba team, and the other was an external member of the research group.

The authors developed the guiding questions used in the interviews and discussed them within the research group. The questions were adapted for the target audience to promote understanding and receptivity among participants (Chart 1).

**Chart 1:** Interview questions according to target audience, São Paulo, São Paulo, Brazil, 2021

Interview questions	
**Children/adolescents**	**Family members/healthcare professionals**
In what situations has the “*Guia Beaba do Câncer*” been used? Support questions, when necessary: ​​when were you admitted? When were you diagnosed? When did you have any questions?	In what situations was the “*Guia Beaba do Câncer*” used?
How was the “*Guia Beaba do Câncer*” used? Support questions, when necessary: did you use it alone? Did the psychologist show it to you? At the hospital’s daycare center?	How was the “*Guia Beaba do Câncer*” used?
What did you learn from the material?	What did you learn through the material?
What was your experience with the “*Guia Beaba do Câncer*” like?	What was your experience with the “*Guia Beaba do Câncer*” like?

The interviews averaged 15 minutes each, resulting in 6.5 hours of final material. They were audio-recorded using a digital device. Data collection was completed using the data saturation technique when recurrent meanings began to emerge in the interviews without new information relevant to the study objectives^(17)^. To ensure this definition, the researchers engaged in systematic deliberations throughout the data collection process. They analyzed each new set of interviews in light of emerging categories. They conducted this process cumulatively and reflexively, comparing the most recent data with previous data to identify repeating patterns and the absence of new analytical elements. When redundancy was observed in the narratives and no new theoretical contributions were identified, the researchers concluded that saturation had been reached. Therefore, it was unnecessary to resend the invitation to the remaining children, adolescents, family members, and professionals who participated in the previous validity study and did not respond to the email. The interviews were transcribed using the automated version of Transcribe by Wreally^®^ software, with corrections made through self-transcription.

### Data analysis

The inductive thematic analysis technique of Braun and Clarke^(18)^ was used for data analysis, with the following stages: 1) Pre-analysis: with a cursory reading of the material, constitution of the *corpus*, formulation and reformulation of hypotheses; 2) Exploration: with the initial categorization and creation of categories through significant expressions; and 3) Interpretation: with the association between the objective and the categorization performed, generating the final analysis. To assist in this process, the ATLAS^®^ software was used. After categorization was complete, the researchers engaged in a collective discussion to deliberate and reach a consensus. It is worth reiterating that inductive thematic analysis was linked to the theoretical framework and that the discourses were discussed in light of Symbolic Interactionism.

## RESULTS

Nine children/adolescents between the ages of 6 and 19 who were undergoing cancer treatment or were in partial or complete remission participated in the study. Seven children/adolescents were interviewed individually without the need for family support, while two were interviewed with their mother. Nine family members of children/adolescents with cancer, as well as nine healthcare professionals who used the educational book, also participated. It is important to note that children/adolescents were not necessarily related to the family members included in the study, and vice versa.

In relation to children/adolescents, the mean age was 12 years (between 6 to 19 years). They were predominantly female (77.7%), from the Southeast and Northeast regions (44%), followed by the South region (11.1%). Most had an elementary education (66.6%), were diagnosed with leukemia (44%), were undergoing their first treatment (77.7%), and had been undergoing treatment for less than two years (88%). Among family members, the mean age was 35 years. There was a predominance of females (66%), and most were from the Southeast region (77%). Most completed high school (44%). As for kinship with the child or adolescent who received the book, mothers were the majority (66%). The most frequent types of cancer among children were neuroblastoma (44%) and leukemia (33%). Among the healthcare professionals, female participants had a mean age of 36 years (100%). They were mainly from the Southeast (55%) and Northeast (44%) regions. Most had training in medicine (33%) or psychology (33%) and had graduate degrees (88%). Additionally, they had more than ten years of experience in the health field (44%). Two categories emerged from the analysis of the interviews, which are described below.

### 1) Reinterpreting words: enhancing children/adolescents’ understanding of cancer with the educational book

In this category, the discourse revealed that interacting with the educational book reinterpreted their experiences with cancer. Reports indicated that technical language that was difficult to understand frequently predominated in communications with healthcare professionals. In the social context, cancer-related stereotypes intensified anxieties about the disease, and the lack of clear, accessible information led to negative emotional reactions. Receiving the material opened up space for new meanings after these experiences.

The book was used countless times to answer questions about terms related to the disease and/or treatment. Through exposure to new experiences, they continued to learn. They used it for themselves and to help others, including patients, family members, and younger siblings. They also used it to disseminate information on their social networks. Children/adolescents reported bringing the educational book to their social groups, such as schools, to help spread information about the disease. They found the visual and written language of the “*Guia Beaba do Câncer*” accessible, fun, and understandable, and appreciated its captivating approach. The book even allowed for group play. Ultimately, negative feelings gave way to greater security and peace of mind. Chart 2 presents speeches from children, adolescents, family members, and healthcare professionals about this topic.

**Chart 2:** Speeches related to the first category, São Paulo, **São Paulo**, Brazil, 2021

Reinterpreting words: enhancing children/adolescents’ understanding of cancer with the educational book
*Whenever the doctor told me a different word, I always went to the book to look it up. I never said, “What do you mean, doctor?!” Because I knew she would explain it her way and it would be perfectly explained in the guide.* (P3) *The doctor said, “You’re going to have chemotherapy”. Me, “What is chemotherapy, my God!? What is neuroblastoma?” I didn’t understand anything, nothing, nothing. I was desperate with what the doctor said.* [...] *I used the guide to understand what the treatment would be like and what part of treatment was like.* (P4) *I understood what cancer is, because I didn’t understand what disease it was.* (P4) *I’d look at it to clear up any doubts. It was more at night that I’d pick up the guide and sit there reading several pages to understand what had happened during the day or during the week.* (P9) *On my Instagram^®^ I make several videos with the guide.* [...] *I explain what chemotherapy is, what cancer is, what neuroblastoma is.* (P4) *My sister, who is five, sometimes we’re talking about something, and she asks questions. Then I take the guide and show it to her.* (P6) *I took it to school, presented it to the teachers, principals, and coordinators.* (P8) *Sometimes a newbie would come in and not know what something was. To avoid explaining things incorrectly, I’d grab the guide and do some research.* (P6) *In the groups, games were played with the guide.* [...] *we learn through play.* (P6) *The word “cancer” is a very strong word.* [...] *many people don’t even like to mention this name, this disease. And the guide shows how complicated it is, but in a more colorful, less painful way.* (P3) *It’s a very cute way of showing something so serious. It’s all illustrative, full of drawings and ways of speaking.* [...] *I was so happy when I received it, the first thing I looked at was my type of cancer.* (P3) *Through the guide, I knew what was happening, I could relax, because I had anxiety attacks before doing the procedures, so it helped me a lot.* (P9) *It’s very rewarding to have this support. When I think of something, I run to the guide, I run to the website. When I want to say something and I don’t know how to explain it, I know Beaba will clarify it for me.* (F01) *In the ward it was like this: I would take a guide and we would look at it, like me holding it and then leafing through it for the child, we would read it.* (HP2) *To try to explain and have tangible material that they could look at, we’d leave it there for them to leaf through. Sometimes I’d go out and leave the guide there for a bit, come back, and ask the child to tell me which part they liked best.* (HP1) *Beaba taught me more about how to talk about difficult things with such truth without having to omit, without having to whine, but without losing tenderness.* (HP1)

### 2) “A watershed moment”: the educational book as a way of connecting family members to the world of cancer and professionals with children

Similar to what the children/adolescents reported, family members said that the educational book helped them reframe their experiences with cancer by providing new interpretations of the phenomenon. They said that their interactions with the cancer diagnosis led to emotional impacts and doubts. However, after receiving and using the guide, they were able to demystify previous symbols. They described this as a “watershed moment”, as their fear and uncertainty turned into confidence and empowerment (Chart 3).

**Chart 3:** Speeches related to the second category, São Paulo, **São Paulo**, Brazil, 2021

“A watershed moment”: the educational book as a way of connecting family members to the world of cancer and professionals with children/adolescents
*The book was a game-changer. I have a diagnosis, I have a vision of the diagnosis, before and after Beaba.* [...] *when we receive the diagnosis, it opens a hole and you go to rock bottom, it seems like you’ll never get out. But you see that the empowerment the child has from knowledge makes you rise with them.* (F6) *Since everything is so new, I had a lot of questions. The names the team uses are completely out of my league.* [...] *the little book is like a dictionary, in a fun and simplified way.* (F8) *The book was sensational because I saw that he* [my son] *knew his reality, what was happening. But at the same time, as a child, he found himself in that more playful, colorful, lively image. My greatest experience with the guide was comfort; I started talking to him more, in a playful way.* (F7) *When he* [his son] *had to go through a bad situation* [...] *I would sit with him beforehand, we would take the guide, he calls it “the boo-boo bear”, and show him the bear. His passion was the bear with the drain; he really identifies with it.* (F4) *From the educational guide, I began to effectively participate in the treatment.* (F6) *People don’t understand that she* [daughter] *is already well. Some people think she lives in the hospital, so we have some information that we can pass on to people.* (F3) *The guide helped me with the nomenclature.* [...] *it’s a serious document, a serious book, which talks in a lighthearted way, but about serious matters.* (F7) *I say “guide”, but Beaba is much more than just a role. He was a gateway to many other connections. He was a passport to the world; a universe I had no idea existed and which I continue to discover.* (F1) *In 2016, Beaba sent a box full of guides. I took all those guides to the hospital in Campina Grande. My God, those children were so enlightened, there’s no other word for it. It was incredible, it was incredible. And then I started using the guide in the outpatient clinic.* (HP2) *I usually sit down to read as if I were telling a story to a child.* (HP8) *Sometimes when we’re discussing something and there’s a word they don’t know the meaning of, I say, “Let’s see everyone at Beaba”. And we really use it like a dictionary, right? I think that’s it, in the most varied ways that come up.* (HP6) *In the first contact with the patient, with the children, to break down some of the fear, so to speak, that they have of talking to professionals, with people they don’t know, then show them the guide, in a playful way talk to them about some things that happen in the treatment and even about my work there, with them, what the nutritionist does.* (HP7)

Family members indicated that they primarily used the educational book to clarify doubts, especially regarding unfamiliar words, understanding diagnosis and treatment, feeling empowered, participating in care, and disseminating information to their children, other family members, parents of children with cancer, and their social groups. The book’s crucial role in communicating with children/adolescents is noteworthy. It uses playful language, colors, and drawings, as well as clear and engaging writing to convey challenging information. Chart 3 presents statements from family members and healthcare professionals about this category.

## DISCUSSION

In this study, we observed that interacting with the educational book provided new meaning to the experiences of children/adolescents with cancer. This interaction led to the acquisition of new knowledge and a reduction in negative emotional reactions to the disease. These results corroborate the first premise of Symbolic Interactionism, which states that individuals act and think about a given phenomenon according to the meaning it holds for them^(14)^. Here, cancer has negative meanings, debt market, which were explained in a fun way with the “*Guia Beaba do Câncer*”.

Communicating with children/adolescents is challenging in clinical practice. The educational book facilitates connection between professionals and this audience. The literature indicates that healthcare professionals tend to communicate with this audience in a technical way, using clinical terminology that is difficult to understand. Otherwise, they do not communicate with the child at all and relay the information to the family instead. This can result in children having little knowledge about their clinical condition and forming their own beliefs, which can lead to negative emotional reactions^(9,19,20)^. In this study, the educational book played a role in reinterpreting the phenomenon and empowering children about their clinical conditions, as seen in the speech: “*The doctors always talk to us, not in a cute way like it’s mentioned here* (educational book). *They explain things in their own way*” (P3).

Families are crucial in the process of communicating with children/adolescents. According to Symbolic Interactionism, an action is governed by the interpretation of a phenomenon^(14)^. Similar to other studies, this study observed the difficulty families have communicating with children/adolescents, sometimes omitting and/or limiting information under the guise of protection^(7,21-23)^. However, family members reinterpreted and facilitated this process by using the educational book as a form of support in communication. Providing children/adolescents with information allows them to have greater control over their condition, reducing stress and increasing their freedom of expression^(10)^. Furthermore, this study demonstrated that the benefits for children/adolescents are mutual for families, acting as a mirror of reactions.

The discussed results also reflect the experiences of families who often used the book because they did not understand the information they received. A cross-sectional study of 209 family members of children with cancer in Iran revealed their desire for clinical and emotional support, as well as guidance on organizing care^(8)^. Even though the educational book focuses on keywords that families encounter in their daily lives with cancer, these needs are intrinsically met. The information acts synergistically because understanding the clinical picture can reduce emotional demands.

To fill the information gap about childhood cancer, the public tends to use technological resources, such as the Internet. A Peruvian study of 365 parents and caregivers of children with cancer found that 72.8% obtained information online^(24)^. Considering that a multicenter investigation analyzing information about childhood cancer on websites demonstrated low quality, accuracy, adequacy, and comprehension, this rate may be worrying. This may have the opposite effect, leading to greater distress^(25)^.

The educational book can act in this context, considering that is accurate in its creation and validity^(12)^, with reliable information, easy to access and understand, as expressed in the speech: *“We use the internet to access more, more robust content. But there* (the educational book) *I had a wealth of information that satisfied my needs”* (F8). The aforementioned Peruvian study found that 53.8% of parents indicated a willingness to use printed materials^(24)^, which supports the results described here. Furthermore, the book was available in print and online, providing access to parents seeking remote information.

In the context of symbolic interactionism, life experiences include moments called epiphanies, which leave a profound mark on individuals’ lives^(14)^. These moments can occur with a cancer diagnosis or with negative communication from professionals about the clinical condition. According to Herbert Blumer, human beings do not respond to the world as it is, but rather to the reality they define for themselves^(14)^. When children, adolescents, and their families define cancer as a devastating life situation, they may become so immersed in this context that they fail to see the possibilities. A lack of information can also be a source of distress. A cross-sectional study of 108 parents of children with cancer in Israel found that 45% had negative outlooks on the future^(7)^.

Essential communication strategies capable of reframing these experiences include the “*Guia Beaba do Câncer*”, which made information more understandable, reducing insecurity and other negative reactions while enhancing the empowerment of the patient-family dyad. According to Symbolic Interactionism, phenomena are dynamic and can be defined and redefined through experience^(14)^. As seen in this study, the children/adolescents reinterpreted the words, enabling the family to connect better. This enabled promising new interactions. The following statement stands out: “*From the guide, I saw how important information is; it strengthens us above all*” (F6).

Based on clear information, it is noteworthy that the child/adolescent and their family begin the process of transmitting it to their surroundings. In the case of children/adolescents, their interaction with their siblings is noteworthy, and the guide provides an opportunity for joint interaction and learning. This interaction is potentially valuable because literature indicates that siblings of children with cancer tend to be overlooked, receiving less care and attention to their needs^(7,10,23)^. Another aspect is that children/adolescents used the guide on social media and in schools. These are moments of interaction that can lead to a public impact and reduce the stigma of cancer by disseminating information. Family members also indicated that they used the guide with other extended family members, which stimulated collective understanding. They also used it with other families who have experienced cancer, which strengthened the dissemination of information.

This exchange of information in a social environment aligns with the second premise of symbolic interactionism, which states that the meaning of phenomena can be modified and expanded through human interaction with their environment^(14)^. Thus, children/adolescents expand their knowledge of their condition and spread it to other groups, who then reinterpret it. The same is seen with families, who benefit mutually.

From an interactionist perspective, the third premise is that interpretations are shaped by new life experiences^(14)^. This aspect is evident in this study, as families emphasize the continued use of the guide due to new care demands related to cancer. One example is frequent hospitalizations in children undergoing active treatment, which create a vicious cycle of new information and difficulty understanding^(11,19)^.

Qualitative research, conducted in Malaysia with parents of children with cancer, demonstrated reports of difficulty in acquiring new clinical information, especially when it was communicated only once, as well as a desire for material that could clearly record it^(11)^. The guide addressed this need and served as a tool for family support, as seen in the speech: “*I even carry the guidebook in my bag. I go to the hospital and it’s with me*” (F8).

Written and visual language were essential to achieving the aforementioned benefits. The clarity, objectivity, and humor of the written texts were especially highlighted by the group of children/adolescents. Objectivity and clarity were emphasized as essential aspects of written language, as they avoid long, detailed sentences that encourage distraction and make reading tiring. In visual language, illustrations, colors, and playfulness were mentioned, making the content tangible and the experience enjoyable^(26)^.

According to symbolic interactionism, meanings begin to be constructed when awareness of a phenomenon is achieved through reflection and interpretation^(14)^. In the case of children, adapting materials to attract them is necessary to achieve this process. Here, we opted for play, the basic language of this audience. Based on the framework of children’s essential needs, it is necessary to respect individual differences and promote developmentally appropriate experiences^(27)^. In this case, the material served a playful function, contributing to enjoyment and redefining the illness. It also served an educational function, demystifying the disease and its treatment while respecting developmental milestones^(28)^.

The use of reading as a therapeutic resource intertwined with care is called bibliotherapy. This concept was mentioned repeatedly by the healthcare professionals who participated in this study. Supported by children’s literature, this technique uses different languages to broaden the experiences of hospitalized children and foster new interpretations of reality. Contributing to this understanding is the critical function of children’s literature, which provides children with a perspective that recognizes their surrounding context^(29)^. Notably, children/adolescents indicated that they used the book in games, which became a way of learning while having fun. This has potential for child development amid the experience of cancer^(10)^.

A similar study conducted in Japan reported on the process of creating an educational book to discuss cancer with children. The results of the Japanese study corroborate the present study’s findings. Professionals and family members indicated the material’s benefits for communication and accessibility for children, which increased their knowledge of the disease^(22)^. However, the Japanese book was limited to disseminating information to children only, whereas the “*Guia Beaba do Câncer*” can be used by children, families, and healthcare professionals alike, thereby advancing the boundaries of knowledge.

Historically, healthcare services have been organized in a paternalistic way, hindering open communication between professionals and patients. This context causes children, adolescents, and their families to feel uncomfortable asking healthcare professionals questions^(30)^. In light of Symbolic Interactionism, action can occur in the “manifest” dimension, with the externalization of sensations, and in the “covert” dimension, with internal experience^(14)^. Taking the two aforementioned aspects into consideration, it is possible that families do not feel comfortable asking professionals questions, instead keeping their doubts to themselves. Additionally, professionals may not dedicate sufficient time to thoroughly clarifying those doubts. It is worth noting that the “*Guia Beaba do Câncer*” is supplementary to professional information, not a substitute. Healthcare providers can use it as a communication tool, but they must spend adequate time with children, adolescents, and their families^(11)^.

In the future, implementation studies are needed to enable the applicability of the “*Guia Beaba do Câncer*” in different contexts within the healthcare network and to assess its use for the general public, not just patients and families affected by cancer, in order to demystify stigmas surrounding the disease. Furthermore, although this study included participants from different parts of Brazil, it is necessary to analyze whether semantic adaptations will be necessary for different cultural groups to ensure culturally responsive care.

### Study limitations

This study has several limitations. First, the majority of participants resided in southeastern Brazil, which may have restricted the diversity of cultural and regional experiences regarding the investigated phenomenon. Since data collection was conducted exclusively online, family members had to be literate, which may have excluded social groups with limited digital access, functional literacy, or difficulty reading the educational material. These factors pose barriers to including different perspectives, particularly those of populations experiencing greater social vulnerability. The educational book was given to patients at a time of emotional vulnerability, which may have positively influenced their perceptions and generated emotional bonds with the material. Although these bonds are legitimate, they may have subjectively impacted participants’ responses. Additionally, including participants who were already familiar with the educational material from the previous validity stage limited the diversity of perceptions and restricted the generalization of the results. However, this approach ensured greater depth in the analysis and continuity in the investigative process.

### Contribution to nursing

This study contributes to the field of nursing by demonstrating the importance of incorporating playful, educational resources into care delivery. The “*Guia Beaba do Câncer*” is a potential strategy for conveying age-appropriate information in a clear, accessible, and welcoming manner. This resource can be used during interactions between children, their families, and healthcare professionals. Furthermore, the material shows promise for nursing practice because it addresses three fundamental pillars of pediatric nursing: (1) Children/adolescents’ rights, especially the right to receive information; (2) Respect for children’s essential needs, recognizing their unique experiences; and (3) Family-centered care, by promoting the empowerment of family members in the care process. Finally, this study highlights the book’s potential as a soft care technology, strengthening communication between nurses, children, adolescents, and their families in clinical practice.

## FINAL CONSIDERATIONS

From the perspective of children, adolescents, family members, and healthcare professionals, the “*Guia Beaba do Câncer*” presents a playful and accessible language, promoting the dissemination of knowledge and demystifying challenging information about cancer. A reduction in negative reactions was observed, in addition to promoting participant empowerment and strengthening interpersonal connections in the context of childhood cancer.

## Data Availability

The research data are available only upon request.
