# Supplementary material for: Reinterpreting words and making connections: an experience with an educational book about childhood cancer
Source: Rev Bras Enferm. 2026 Jul 10;79:e20240571. doi: 10.1590/0034-7167-2024-0571 (PMC13363302; doi:10.1590/0034-7167-2024-0571)
Supplement: Translation

# Translation: Reinterpretando palavras e fazendo conexões: Reinterpretar palabras y crear conexiones: una experiencia con un libro educativo sobre el cáncer infantil

**DOI:** 10.1590/0034-7167-2024-0571pt

**Authors:** Simone Lehwess Mozzilli, Danton Matheus de Souza, Milena Santos de Oliveira, Marina de Góes Salvetti

**Affiliations:** I Universidade de São Paulo. São Paulo, São Paulo, Brasil; II Instituto Beabá. São Paulo, São Paulo, Brasil; III Universidade Federal de São Paulo. São Paulo, São Paulo, Brasil

**Keywords:** Comunicação em Saúde, Tecnologia da Informação, Neoplasias, Enfermagem Pediátrica, Enfermagem Familiar.

## Abstract

**Objetivos:**

compreender a experiência de crianças/adolescentes em tratamento oncológico, familiares e profissionais de saúde com o livro educativo “Guia Beaba do Câncer”.

**Métodos:**

estudo exploratório, qualitativo, sob a óptica do Interacionismo Simbólico. A coleta de dados foi conduzida *online* com nove crianças/adolescentes com câncer, nove familiares e nove profissionais de saúde. Foram conduzidas entrevistas semiestruturadas, gravadas e transcritas, analisadas pela técnica de análise temática indutiva.

**Resultados:**

emergiram duas categorias: 1) Reinterpretando palavras: potencializando a compreensão das crianças/adolescentes sobre o câncer com o livro educativo; e 2) “Um divisor de águas”: o livro educativo como uma forma de conexão dos familiares ao mundo do câncer e dos profissionais com crianças/adolescentes.

**Considerações Finais:**

o livro educativo foi reconhecido por sua linguagem lúdica e acessível, promovendo a ressignificação de experiências e símbolos relacionados à vivência do câncer, além de desmistificar informações, reduzir reações negativas e difundir conhecimentos.

## INTRODUÇÃO

Nos últimos anos, evidenciou-se transição epidemiológica na área infantojuvenil, com o aumento do número de doenças crônicas não transmissíveis^(1)^. Neste perfil, chama a atenção o câncer infantojuvenil, que se tornou um problema de saúde pública global. A Organização Mundial da Saúde estima que, a cada ano, mais de 400 mil crianças, entre 0 e 19 anos incompletos, são diagnosticadas com câncer^(2)^. No Brasil, estima-se que, para cada ano do triênio de 2023 a 2025, mais de sete mil crianças sejam diagnosticadas com câncer, com um risco estimado de 134,8 por milhão de crianças/adolescentes^(3)^. O público infantojuvenil acometido pelo câncer tem cerca de 80% de possibilidade de alcançar a cura se diagnosticado precocemente e tratado em centros especializados^(1)^. Todavia, mais de 50% dos diagnósticos ocorrem em estágios avançados do câncer, especialmente pela falta de informação da comunidade leiga sobre o quadro clínico^(4)^.

Estudos prévios relataram inúmeros impactos do diagnóstico e curso do câncer em crianças, adolescentes, suas famílias e grupos sociais em que estão inseridos^(5-7)^. A experiência circunda mudanças pessoais, relacionais e ambientais. Há rompimento das expectativas futuras e medo da morte, além de interrupção dos sonhos, desejos, ambições e realizações, com reações emocionais como ansiedade, depressão e sensação de impotência^(5-8)^. Diversos estudos mostram que a falta de informações está relacionada a esses desfechos negativos^(5-9)^. Neste contexto, torna-se necessário um olhar atento ao câncer infantojuvenil e à disseminação de informações claras e otimistas sobre esta condição.

Estudo transversal, realizado na Indonésia, com 100 familiares de crianças com câncer, observou falta de informações e falhas no suporte relacionado à doença e ao seu curso de cuidados, com ênfase em informações clínicas, o que dificulta a compreensão e favorece o aumento das reações emocionais^(4)^. No Brasil, investigação qualitativa com 27 familiares de crianças com câncer demonstrou o desejo de informações acessíveis e compreensíveis, sendo uma forma de alívio e empoderamento no cuidado à criança^(5)^.

Para disseminar informações claras e acessíveis, é importante que os profissionais de saúde utilizem tecnologias leves de cuidado na comunicação^(10)^. Uma dessas possibilidades é o uso de livros educativos, enfoque deste estudo, que permite a explicação do diagnóstico, procedimentos, exames e terapêutica adotada, favorecendo o enfrentamento saudável e a satisfação da criança, adolescente e família com o cuidado^(11)^.

O livro educativo denominado “Guia Beaba do Câncer” foi desenvolvido para oferecer informações claras e acessíveis, desmistificar o câncer infantojuvenil, e melhorar a qualidade da comunicação em saúde. O processo de criação do “Guia Beaba do Câncer” contou com a participação ativa de crianças/adolescentes em tratamento oncológico, seus familiares, profissionais de saúde e especialistas em tecnologia da informação e comunicação. Em 2024, o conteúdo do livro educativo foi validado com sucesso, na perspectiva de crianças, adolescentes, famílias e profissionais de saúde^(12)^.

A produção deste livro educativo está alinhado às prioridades emergentes de pesquisa na área infantojuvenil para esta década, destacando-se estudos que: compreendam o uso de intervenções para a autogestão de quadros clínicos; otimizem a comunicação e compartilhamento de informações no cuidado; avaliem diferentes programas de apoio após o diagnóstico de doenças crônicas; e desenvolvam estratégias para auxiliar a família na tomada de decisão ao longo do cuidado à criança e ao adolescente^(13)^. Essas prioridades se interligam com os aspectos supracitados como base para a construção deste estudo, que parte das seguintes inquietações: como crianças, adolescentes e famílias compreendem o uso de um livro educativo sobre o câncer infantojuvenil na sua experiência com a doença? Como os profissionais de saúde utilizam o livro educativo?

Para responder à questão de pesquisa, o referencial teórico do Interacionismo Simbólico de Herbert Blumer^(14)^ foi utilizado. Esse referencial indica que, para alcançar a compreensão de um fenômeno na vida de um indivíduo, deve-se integrar às suas interações vivenciadas na dinâmica social o seu discurso. Por exemplo, neste estudo, as crianças, adolescentes, famílias e profissionais de saúde interageme consigo e entre si, com seu contexto social, com o câncer e com o livro educativo, e todos esses fatores influenciam suas experiências.

## OBJETIVOS

Compreender a experiência de crianças/adolescentes em tratamento oncológico, familiares e profissionais de saúde diante do uso do livro educativo “Guia Beaba do Câncer”.

## MÉTODOS

### Aspectos éticos

O presente estudo é parte de projeto maior, e os dados são provenientes da dissertação de mestrado intitulada “Validação de livro educativo para famílias com crianças ou adolescentes em tratamento oncológico e análise da experiência”^(15)^. O estudo foi aprovado pelo Comitê de Ética em Pesquisa da Escola de Enfermagem da Universidade de São Paulo. O Termo de Consentimento Livre e Esclarecido e o Termo de Assentimento foram obtidos de todos os participantes *online*. Respeitaram-se as diretrizes éticas nacionais e internacionais. Os discursos foram identificados pelas letras P (paciente-criança e/ou adolescente), F (familiar) ou PS (profissional de saúde), seguidas de um número com base na ordem de inclusão no estudo: P1, F1, PS1...

### Referencial teórico

Para compreensão das experiências das crianças, adolescentes, familiares e profissionais de saúde, optou-se por analisar os dados à luz do referencial teórico do Interacionismo Simbólico de Herbert Blumer^(14)^. Neste referencial teórico, parte-se do pressuposto de que as percepções são geradas por intermédio das interações da dinâmica social as quais os indivíduos estão imersos. Os indivíduos se envolvem em interações sociais, influenciadas por seu contexto mais amplo, que provocam ações que são interpretadas, moldando suas percepções e significados de um fenômeno específico que, no caso deste estudo, é a experiência com o uso do “Guia Beaba do Câncer”. O Interacionismo Simbólico possui três premissas que foram utilizadas: 1) As ações dos indivíduos são resultadas dos significados que um fenômeno possui para si; 2) O significado do fenômeno emerge das interações do indivíduo no seu meio coletivo; e 3) Os significados de um fenômeno podem ser modificados a partir de novas interações em sua dinâmica social.

### Delineamento do estudo

Estudo descritivo exploratório, de abordagem qualitativa. Para a sua redação, utilizou-se o *COnsolidated criteria for REporting qualitative Research* ^(16)^.

### Cenário do estudo e recrutamento

O estudo foi conduzido de forma remota, pela plataforma *Zoom* ^®^, entre dezembro de 2020 e janeiro de 2021. A abordagem *online* foi escolhida visando à possibilidade de inclusão de crianças, adolescentes, familiares e profissionais de saúde de diferentes locais do Brasil que tivessem tido contato com o livro educativo, somada à declaração da pandemia de COVID-19, que reduziu o número de indivíduos externos que circulavam nos hospitais oncológicos da região de atuação dos pesquisadores.

A população do estudo foi composta por crianças/adolescentes que vivenciaram o câncer na infância ou juventude, além de seus familiares e profissionais de saúde. Incluíram-se crianças/adolescentes com idade igual ou superior a 4 anos, em tratamento oncológico, em fase de manutenção, remissão parcial ou total da doença. Para os familiares, foram incluídos indivíduos com 18 anos ou mais pertencentes ao núcleo familiar da criança e autodeclarados alfabetizados. Quanto aos profissionais de saúde, foram incluídos aqueles com experiência mínima de 24 meses na assistência direta a crianças/adolescentes com câncer. Em comum, todos deveriam ter utilizado e/ou ter tido contato com o livro educativo “Guia Beaba do Câncer” e ter acesso à internet. Foram excluídos do estudo pessoas com dificuldade de comunicação, prejuízo cognitivo ou doenças incapacitantes.

No período do estudo, o “Guia Beaba do Câncer” estava em sua segunda edição. A primeira edição contou com 2.250 exemplares, distribuídos diretamente aos hospitais oncológicos de todo o Brasil. A segunda edição contou com 4.250 exemplares, distribuídos diretamente aos hospitais e a 163 pacientes/famílias e profissionais que solicitaram o guia pelo e-mail da instituição “Beaba”, e o receberam pelo correio, tendo seus respectivos e-mails cadastrados. No estudo principal, mencionado anteriormente, foram enviados por e-mail convites a esses 163 e-mails cadastrados para participar do estudo de validação do guia^(12)^.

Neste estudo, optou-se por convidar os participantes que já haviam integrado a etapa prévia de validação de conteúdo, a fim de garantir a continuidade do processo investigativo, por meio da coleta de dados qualitativa com indivíduos que já estavam familiarizados com o objeto de pesquisa. Assim, foram convidados, por e-mail, para participar, 12 crianças/adolescentes, 20 familiares e 29 profissionais de saúde que participaram do estudo prévio de validação^(12)^. Contudo, apenas nove crianças/adolescentes, nove familiares e nove profissionais de saúde que utilizaram o livro educativo “Guia Beaba do Câncer” durante o tratamento para o câncer responderam ao convite e aceitaram participar, constituindo a amostra do presente estudo.

### Coleta e organização dos dados

Inicialmente, os pesquisadores construíram e a validaram o livro educativo “Guia Beaba do Câncer”^(12)^. O livro faz parte dos materiais educativos da Organização da Sociedade Civil de Interesse Público - Beaba, que atua em todo o Brasil, formada por pacientes, familiares e profissionais de saúde, e que atua disseminando informações complexas em linguagem clara, acessível e otimista. O livro foi construído em ordem alfabética, na forma de um dicionário de termos oncológicos, definidos de modo acessível, com palavras como “hemácias”, “neutropenia” e “líquor”, esclarecidas de modo claro e bem-humorado. A Figura 1exemplifica a ilustração do livro.

**Figura 1:** Livro educativo “Guia Beaba do Câncer”, São Paulo, São Paulo, Brasil, 2022

O livro educativo é divulgado pelas redes sociais da organização, e pode ser acessado por crianças, adolescentes e familiares, de forma gratuita, após pedido de doação no site da instituição. O material também é disponibilizado impresso em instituições de saúde vinculadas à atuação da organização.

Para a coleta de dados, foram realizadas entrevistas semiestruturadas, pré-agendadas e gravadas pela plataforma *Zoom* ^®^, realizadas, de forma individual, com a criança ou adolescente, sua família ou profissional de saúde. As entrevistas foram conduzidas por duas pesquisadoras, do sexo feminino, graduadas em psicologia, com experiência na condução de estudos qualitativos e treinadas previamente pela pesquisadora principal. Uma psicóloga fazia parte da equipe do Beaba, e outra era uma externa, membro do grupo de pesquisa.

O roteiro de questões norteadoras utilizado nas entrevistas foi elaborado pelas autoras e discutido no grupo de pesquisa. As perguntas foram adaptadas segundo público-alvo, visando facilitar a compreensibilidade e receptividade dos participantes (Quadro 1).

**Quadro 1:** Perguntas utilizadas nas entrevistas segundo público-alvo, São Paulo, São Paulo, Brasil, 2021

Perguntas da entrevista	
**Crianças/adolescentes**	**Familiares/profissionais de saúde**
Em quais situações o “Guia Beaba do Câncer” foi usado? Perguntas de suporte, quando necessário: quando você internou? Quando foi diagnosticado? Quando teve alguma dúvida?	Em quais situações o “Guia Beaba do Câncer” foi utilizado?
Como o “Guia Beaba do Câncer” foi usado? Perguntas de suporte, quando necessário: você usou sozinho? A psicóloga te mostrou? Na escolinha do hospital?	Como o “Guia Beaba do Câncer” foi utilizado?
O que você aprendeu com o material?	O que você aprendeu através do material?
Como foi para você a experiência com o “Guia Beaba do Câncer”?	Como foi para você a experiência com o “Guia Beaba do Câncer”?

As entrevistas duraram em média 15 minutos, com 6,5 horas de material final. Foram gravadas por meio de áudio, com dispositivo digital. A coleta de dados foi finalizada com base na técnica de saturação dos dados, considerada atingida quando as entrevistas passaram a apresentar recorrência de significados, sem o surgimento de novas informações relevantes para os objetivos do estudo^(17)^. Para assegurar essa definição, os pesquisadores realizaram deliberações sistemáticas ao longo da coleta, analisando cada novo conjunto de entrevistas à luz das categorias emergentes. Esse processo foi conduzido de forma cumulativa e reflexiva, comparando os dados mais recentes com os anteriores, a fim de verificar a repetição de padrões e a ausência de novos elementos analíticos. Quando se observou a redundância nas narrativas e nenhuma nova contribuição teórica foi identificada, os pesquisadores entraram em consenso que a saturação havia sido alcançada. Dessa forma, não se fez necessário reenviar o convite às demais crianças/adolescentes, familiares e/ou profissionais que participaram do estudo de validação anterior e não haviam retornado o e-mail. Para transcrição das entrevistas, foi utilizado o *software Transcribe by Wreally* ^®^, com a versão automatizada da ferramenta, com correções realizadas através de autotranscrição.

### Análise de dados

Para a análise de dados, utilizou-se a técnica de análise temática indutiva de Braun e Clarke^(18)^, com as etapas de: 1) Pré-análise: com a leitura flutuante do material, constituição do *corpus*, formulação e reformulação das hipóteses; 2) Exploração: com a categorização inicial e criação de categorias através das expressões significativas; e 3) Interpretação: com a associação entre o objetivo e a categorização realizada, sendo gerada a análise final. Para o auxílio nesse processo, utilizou-se o *software* ATLAS^®^. Ao término da categorização, houve uma discussão coletiva entre os pesquisadores para uma deliberação e consenso. Reitera-se que a análise temática indutiva foi associada ao referencial teórico, com uma discussão dos discursos à luz do Interacionismo Simbólico.

## RESULTADOS

Participaram do estudo nove crianças/adolescentes com idades entre 6 e 19 anos, em tratamento oncológico, manutenção, remissão parcial ou total da doença e que fizeram uso do livro educativo. Sete crianças/adolescentes foram entrevistadas individualmente, sem a necessidade de acompanhamento familiar, enquanto duas crianças realizaram a entrevista em conjunto à sua mãe. Também participaram nove familiares de crianças/adolescentes com câncer e nove profissionais de saúde que fizeram uso do livro educativo. Destaca-se que não necessariamente as crianças/adolescentes possuíam parentesco com os familiares incluídos no estudo e vice-versa.

Em relação às crianças/adolescentes, a idade média foi de 12 anos (entre seis e 19 anos), sendo do sexo feminino (77,7%), procedentes das regiões Sudeste e Nordeste (44%), seguidas da região Sul (11,1%), com ensino fundamental (66,6%), diagnóstico de leucemia (44%), em seu primeiro tratamento (77,7%) e com duração menor que dois anos (88%). Já entre os familiares, a idade média foi de 35 anos, prevalecendo o sexo feminino (66%), ser da região Sudeste (77%) e com ensino médio completo (44%). Em relação ao parentesco com a criança ou adolescente que recebeu o livro, o predomínio foi de mães (66%), e os tipos de câncer mais frequentes entre as crianças foram neuroblastoma (44%) e leucemia (33%). Entre os profissionais de saúde, observaram-se participantes do sexo feminino (100%) com idade média de 36 anos, principalmente das regiões Sudeste (55%) e Nordeste (44%), com formação na área da medicina (33%) ou psicologia (33%), com pós-graduação (88%) e mais de dez anos de experiência na área da saúde (44%). A partir da análise das entrevistas, emergiram duas categorias, descritas abaixo.

### 1) Reinterpretando palavras: potencializando a compreensão das crianças/adolescentes sobre o câncer com o livro educativo

Nesta categoria, observou-se nos discursos que a interação com o livro educativo proporcionou a ressignificação das suas experiências com o câncer. Observaram-se relatos de que, frequentemente, nas comunicações com os profissionais de saúde, a linguagem técnica, de difícil compreensão, predominava. No contexto social, os estereótipos relacionados ao câncer potencializavam angústias sobre a doença, e a ausência de informações claras e acessíveis levava a reações emocionais negativas. Após a entrega do material, essas experiências abriram espaço para novos significados.

O livro foi utilizado inúmeras vezes, quando houve dúvida de termos relacionados à doença e/ou ao tratamento, com aprendizados contínuos, ao serem expostos a novas experiências. Utilizaram para si e/ou para ajudar outros, como pacientes e/ou familiares, a exemplo dos irmãos mais novos, e para difusão de informações nas suas redes sociais. As crianças/adolescentes relataram levar o livro educativo para seus grupos sociais, como nas escolas, para ajudar a disseminar as informações sobre a doença. A linguagem visual e escrita do “Guia Beaba do Câncer” foi vista como acessível, divertida e compreensível, com abordagem que prende a atenção. O livro permitia, até mesmo, brincadeiras coletivas. Ao fim, os sentimentos negativos abriram espaço para maior segurança e tranquilidade. O Quadro 2 traz discursos das crianças/adolescentes, familiares e profissionais de saúde sobre esta categoria.

**Quadro 2:** Discursos referentes à primeira categoria, São Paulo, **São Paulo**, Brasil, 2021

Reinterpretando palavras: potencializando a compreensão das crianças/adolescentes sobre o câncer com o livro educativo
*Sempre que a médica me falava alguma palavra diferente, eu sempre ia no livro procurar. Eu nunca dizia assim: “Como assim, doutora?!” Porque eu sabia que ela ia explicar da forma dela e no guia ia estar bem direitinho.* (P3) *A médica falou: “Você vai tomar quimioterapia”. Eu: “O que é quimioterapia, meu Deus!? O que é neuroblastoma?”. Eu não entendia nada, nada, nada. Eu ficava desesperada com o que a médica falava.* [...] *eu utilizei o guia para entender como seria o tratamento e como foi parte do tratamento*. (P4) *Eu entendi o que é o câncer, porque eu não entendia, que doença que era.* (P4) *Eu olhava para tirar dúvida. Era mais de noite que eu pegava o guia e ficava lá lendo várias páginas para entender o que aconteceu durante o dia ou durante a semana.* (P9) *No meu Instagram^®^, eu faço vários vídeos com o guia.* [...] *eu explico o que é quimioterapia, o que é câncer, o que é neuroblastoma.* (P4) *A minha irmã, que tem cinco anos, às vezes a gente está conversando alguma coisa, ela fica perguntando. Aí eu pego o guia e mostro para ela.* (P6) *Eu levei para a escola, apresentei para as professoras, diretoras e coordenadoras*. (P8) *Às vezes, chegava algum novato que não sabia o que era alguma coisa. Para não explicar errado, eu pegava o guia, dava uma pesquisada.* (P6) *Nos grupos, eram feitas brincadeiras com o guia.* [...] *aprendemos com a brincadeira*. (P6) *A palavra “câncer” é uma palavra muito forte*. [...] *muitas pessoas não gostam nem de falar esse nome, essa doença. E no guia, mostra que é complicado, mas mostra de uma forma mais colorida, menos dolorida.* (P3) *É uma forma muito bonitinha de mostrar uma coisa tão séria. Todo ilustrativo, cheio de desenho, formas de falar.* [...] *eu fiquei tão feliz quando recebi. A primeira coisa que fui olhar foi sobre o meu tipo de câncer.* (P3) *Pelo guia, eu sabia o que estava acontecendo, dava para relaxar, porque eu tinha crises de ansiedade antes de fazer os procedimentos, então ele me ajudou muito.* (P9) *É muito gratificante ter esse suporte. Quando eu penso em alguma coisa, eu corro para o guia, eu corro para o site. Quando eu quero falar alguma coisa e eu não sei explicar, eu sei que o Beaba vai me clarear isso.* (F01) *Na enfermaria, era assim: eu levava um guia e a gente ficava vendo, tipo eu segurando, e aí folheando para a criança, a gente ia lendo.* (PS2) *Para tentar explicar e ter um material inclusive palpável que eles pudessem olhar, a gente deixava ali com eles, eles folheavam. Às vezes, eu saia e deixava o guia lá um pouco, voltava, pedia para que a criança me dissesse qual a parte que mais gostou.* (PS1) *O Beaba me ensinou mais a como falar de coisas difíceis com tamanha verdade sem precisar omitir, sem precisar fazer mimimi, mas sem perder a ternura.* (PS1)

### 2) “Um divisor de águas”: o livro educativo como uma forma de conexão dos familiares ao mundo do câncer e dos profissionais com crianças/adolescentes

De forma semelhante à experiência referida pelas crianças/adolescentes, os familiares indicaram que o livro educativo auxiliou na ressignificação das suas experiências com o câncer, com novas interpretações sobre o fenômeno vivenciado. Os familiares indicaram que a forma que interagiam com o diagnóstico de câncer levou a inúmeros impactos emocionais e muitas dúvidas. Contudo, ao receber e utilizar o guia, conseguiram desmistificar os símbolos prévios, fazendo analogia a um “divisor de águas”, pois a dificuldade e o medo abriram espaço para confiança, maior segurança e empoderamento (Quadro 3).

**Quadro 3:** Discursos referentes à segunda categoria, São Paulo, **São Paulo**, Brasil, 2021

“Um divisor de águas”: o livro educativo como uma forma de conexão dos familiares ao mundo do câncer e dos profissionais com crianças/adolescentes
*O livro foi um divisor de águas. Eu tenho um diagnóstico, tenho uma visão do diagnóstico antes e depois do Beaba.* [...] *quando a gente recebe o diagnóstico, você abre um buraco e você vai até o fundo do poço, parece que não vai sair nunca de lá. Mas você vai vendo que o empoderamento que a criança tem a partir do conhecimento faz com que você levante junto com ela.* (F6) *Como tudo é muito novo, eu tive várias dúvidas. Os nomes que a equipe usa é totalmente fora da minha realidade.* [...] *o livrinho é como se fosse um dicionário, de forma lúdica e simplificada.* (F8) *O livro foi sensacional, porque eu vi que ele* [filho] *sabia da realidade dele, do que estava acontecendo. Mas ao mesmo tempo, como criança, ele se encontrou naquela imagem mais lúdica, colorida, animada. A minha maior experiência com o guia foi dar conforto. Comecei a conversar mais com ele, de forma lúdica*. (F7) *Quando ele* [filho] *tinha que passar por uma situação ruim* [...] *eu me sentava com ele antes, a gente pegava o guia, ele chama de “ursinho dodói”, mostrava o ursinho. A paixão dele era o ursinho com dreno, ele se identifica muito.* (F4) *A partir do guia educativo, eu passei a participar efetivamente do tratamento*. (F6) *As pessoas não entendem que ela* [filha] *já está bem. Tem gente que acha que ela mora no hospital, então a gente tem algumas informações que a gente consegue passar para as pessoas*. (F3) *O guia me ajudou com as nomenclaturas*. [...] *é um documento sério, um livro sério, que fala de maneira leve, mas de coisas sérias*. (F7) *Eu falo “guia”, mas o Beaba é muito além do papel. Ele foi uma porta de entrada para fazer muitas outras conexões. Foi um passaporte para o mundo, um universo que eu não fazia ideia que existia e continuo descobrindo.* (F1) *O Beaba mandou em 2016 uma caixa cheia de guias. Eu levei esses guias todos para o hospital em Campina Grande. Meu Deus do céu, essas crianças ficaram assim, iluminadas, porque não tem outra palavra. Foi incrível, foi incrível. E aí eu comecei usando o guia no ambulatório.* (PS2) *Normalmente, eu sento para ler como se eu estivesse contando uma historinha mesmo para criança.* (PS8) *Às vezes, quando a gente está discutindo alguma coisa e tem uma palavra que eles não sabem o significado, então eu digo: “Vamos ver todo mundo no Beaba”. E a gente usa mesmo como se fosse um dicionário, né!? Acho que é isso, das maneiras mais variadas assim que vai surgindo.* (PS6) *Num primeiro contato com paciente, com as crianças para quebrar um pouco do medo, digamos assim, que eles têm de conversar com os profissionais, com as pessoas que não são conhecidas, então mostrar ali o guia, de uma forma lúdica falar com eles sobre algumas coisas que acontecem no tratamento e até sobre o meu trabalho lá dentro, com eles, o que a nutricionista faz.* (PS7)

Os familiares indicaram que fizeram uso do livro educativo para, principalmente, esclarecer dúvidas, especialmente em casos de palavras desconhecidas, compreendendo o diagnóstico e tratamento, sentindo-se empoderados, participando do cuidado e difundindo as informações para os filhos, familiares, outros pais de crianças com câncer e o grupo social em que estavam inseridos. Chama a atenção o papel crucial do livro na comunicação com as crianças/adolescentes, fazendo uso da linguagem lúdica, com cores e desenhos, além de escrita clara e divertida para transmitir informações desafiadoras. O Quadro 3 traz discursos dos familiares e profissionais de saúde sobre esta categoria.

## DISCUSSÃO

Neste estudo, observou-se que a interação das crianças/adolescentes e familiares com o livro educativo proporcionou ressignificação das suas experiências com o câncer, com a aquisição de novos conhecimentos, levando à redução de reações emocionais negativas perante a vivência da doença. Esses resultados corroboram o Interacionismo Simbólico, que traz em sua primeira premissa que o indivíduo age e pensa sobre um determinado fenômeno de acordo com o significado que esse possui a si^(14)^. Aqui, o câncer denotava significados negativos, marcado de dúvidas, que foram esclarecidas de forma divertida com o “Guia Beaba do Câncer”.

A comunicação com crianças/adolescentes é um desafio na prática clínica, e o livro educativo facilitou a conexão entre profissionais, crianças/adolescentes. A literatura indica que os profissionais de saúde tendem a se comunicar com esse público de forma técnica, com terminologia clínica e de difícil compreensão, ou não se comunicam com a criança, repassando a informação para as famílias. Esse processo leva ao desconhecimento da criança sobre o seu quadro clínico, com o estabelecimento de convicções próprias que podem levar a reações emocionais negativas^(9,19,20)^. Neste estudo, o livro educativo atuou no processo de reinterpretação do fenômeno, empoderando a criança sobre seu quadro clínico, como visto no discurso: “*As médicas, elas sempre falam para a gente, não de uma forma bonitinha como é citado aqui* (livro educativo). *Elas explicam da forma delas*” (P3).

No processo de comunicação com a criança e adolescente, as famílias são cruciais. À luz Interacionismo Simbólico, a interpretação de um fenômeno rege uma ação^(14)^. Aqui, semelhante a outros estudos, observou-se a dificuldade da família de se comunicar com a criança e adolescente, por vezes omitindo e/ou limitando as informações com a crença de proteção^(7,21-23)^. Porém, esse processo foi ressignificado e facilitado com o uso do livro educativo pelos familiares, que usaram o material como forma de apoio na comunicação. A entrega das informações às crianças/adolescentes permite maior controle sobre sua condição, reduzindo a carga de estresse e aumentando a liberdade de expressão^(10)^. Ainda, este estudo demonstrou que os benefícios para a criança e o adolescente são mútuos para a família, como um espelho de reações.

Os resultados discutidos também refletem a experiência da família, que muitas vezes fez uso do livro por não compreender as informações recebidas. Investigação transversal, realizada com 209 familiares de crianças com câncer no Irã, observou o desejo dessas de informações clínicas, emocionais e que auxiliem na sua organização perante o cuidado^(8)^. Por mais que o livro educativo focalize em palavras-chaves que as famílias se deparam no dia a dia com o câncer, de forma intrínseca, essas necessidades são atingidas, pois as informações atuam de forma sinérgica, pois a compreensão do quadro clínico pode reduzir as demandas emocionais.

Para preencher a lacuna de informações sobre o câncer infantojuvenil, o público tende a fazer uso de recursos tecnológicos, como a internet. Em estudo peruano, com 365 pais e cuidadores de crianças com câncer, 72,8% indicaram adquirir as informações pela internet^(24)^. Esse índice pode ser preocupante, considerando que investigação multicêntrica que analisou as informações sobre o câncer infantojuvenil em *websites* demonstrou baixa qualidade, precisão, adequação e compreensão, o que pode ter efeito contrário, levando a uma maior angústia^(25)^.

O livro educativo pode atuar nesse contexto, considerando que possui acurácia em sua criação e validação^(12)^, com informações confiáveis, de fácil acesso e compreensão, como expresso no discurso: “*A gente usa a internet para ter mais conteúdo, mais robusto. Mas ali* (livro educativo) *eu tinha uma série de informações que satisfaziam a minha necessidade*” (F8). No estudo peruano supracitado, 53,8% dos pais indicaram a potencialidade do uso de materiais impressos^(24)^, o que corrobora os resultados aqui descritos. Ainda, o livro foi disponibilizado na forma impressa ou *online*, o que pode permitir que os pais que busquem informações remotas também tenham acesso.

À luz do Interacionismo Simbólico, nas experiências de vida, há momentos denominados de epifanias, que deixam marcas profundas na vida dos indivíduos^(14)^, e isso pode ocorrer no diagnóstico do câncer e em cada comunicação negativa dos profissionais sobre a condição clínica. Hebert Blumer indica que os seres humanos não respondem ao mundo como ele é, mas sim respondem à realidade definida por si^(14)^. Neste caso, quando as crianças/adolescentes e famílias definem o câncer como uma situação devastadora da vida, podem ficar imersos neste contexto, não enxergando as possibilidades, e a falta de informação pode ser mais uma fonte de desgaste. O medo predomina, como visto em investigação transversal com 108 pais de crianças com câncer em Israel, com 45% indicando perspectivas negativas sobre o futuro^(7)^.

Formas de se comunicar capazes de ressignificar essas vivências são essenciais, como o “Guia Beaba do Câncer”, que permitiu que a informação se tornasse compreensível, reduzindo a insegurança e demais reações negativas, potencializando o empoderamento do binômio paciente-família. À luz do Interacionismo Simbólico, os fenômenos são dinâmicos, podendo ser definidos e redefinidos com as experiências^(14)^. Como visto neste estudo, as palavras foram reinterpretadas pelas crianças/adolescentes, e a família conseguiu se conectar melhor. A informação permitiu novas interações promissoras, destacando-se o seguinte discurso: “*A partir do guia, eu vi o quanto a informação é importante; ela nos fortalece acima de tudo*” (F6).

Destaca-se que, a partir das informações claras, a criança/adolescente e família iniciam o processo de transmissão dessas para o seu contexto. No caso das crianças/adolescentes, chama a atenção a sua interação com seus irmãos, sendo o guia um momento de interação conjunta e aprendizado. Esse momento é potencial, uma vez que a literatura indica que os irmãos das crianças com câncer tendem a ser invisibilizados, com menor dedicação de cuidados e atenção às suas necessidades^(7,10,23)^. Outro aspecto é que as crianças/adolescentes fizeram uso do guia em suas redes sociais e nas escolas, momentos de interação com o contexto que podem levar a impactos públicos, reduzindo o estigma do câncer com a difusão das informações. Já os familiares indicaram o uso com outros membros da família extensa, fato potencializador que estimulou a compreensão coletiva, e com outras famílias que compartilham a experiência com o câncer, fortalecendo a difusão das informações.

Essa troca de informações em meio social pode ser associada à segunda premissa do Interacionismo Simbólico, na qual o significado dos fenômenos pode ser modificado e expandido com as interações que o ser humano tem com seu contexto^(14)^. Assim, as crianças/adolescentes expandem seus conhecimentos sobre sua condição, difundindo a outros grupos que a reinterpretam. O mesmo é visto com as famílias, com ganho mútuo.

Em uma perspectiva interacionista, na terceira premissa, observa-se que as interpretações são moldadas com as novas experiências de vida^(14)^, e esse aspecto é visto neste estudo, considerando que as famílias ressaltam a continuidade do uso do guia devido às novas demandas de cuidado relacionadas ao câncer. Um exemplo é com as hospitalizações, frequentes em crianças em fase ativa do tratamento, em que há um ciclo vicioso: novas informações e dificuldades de compreensão^(11,19)^.

Investigação qualitativa, realizada na Malásia com pais de crianças com câncer, demonstrou relatos de dificuldade de aquisição de novas informações clínicas, especialmente quando comunicadas apenas uma vez, desejando algum material que pudesse registrá-las de forma clara^(11)^. Com o guia, foi possível atuar nesta demanda, sendo um instrumento para auxílio familiar, como visto no discurso: “*Inclusive, o guia anda na minha bolsa. Eu vou no hospital, ele está comigo*” (F8).

Para atingir os benefícios supracitados, as linguagens escrita e visual foram essenciais. Na linguagem escrita, foram destacadas a clareza, objetividade e bom humor dos textos, principalmente pelo grupo de crianças/adolescentes. Entre os aspectos essenciais da linguagem escrita, destacam-se a objetividade e a clareza, evitando frases longas e detalhadas que levam à distração e tornam a leitura cansativa. Na linguagem visual, observaram-se menções às ilustrações, às cores e ao lúdico, tornando o conteúdo palpável e a experiência agradável^(26)^.

Para o Interacionismo Simbólico, os significados começam a ser construídos quando há consciência sobre o fenômeno, com reflexão e interpretação^(14)^. No caso das crianças, para atingir esse processo, é necessário adaptar os materiais para atraí-las, e aqui optou-se pelo uso do lúdico, linguagem base deste público. Partindo do referencial das necessidades essenciais das crianças, torna-se necessário respeitar as diferenças individuais e promover experiências adequadas ao seu desenvolvimento^(27)^. Aqui o material apresentou função lúdica, contribuindo para diversão e ressignificação do adoecimento, e também com a função educativa, desmistificando e ensinando sobre a doença e seu tratamento, respeitando seus marcos de desenvolvimento^(28)^.

A utilização da leitura entrelaçada ao cuidado como fonte terapêutica é denominada de “biblioterapia”, mencionada diversas vezes pelos profissionais de saúde que participaram deste estudo. Essa técnica, com o auxílio da literatura infantil, utiliza diferentes linguagens, com o objetivo de ampliar vivências de crianças hospitalizadas, favorecendo novas interpretações da realidade. Contribuindo com esse pensamento, a função crítica da literatura infantil proporciona para a criança um olhar de reconhecimento do contexto à sua volta^(29)^. Chama a atenção que as crianças/adolescentes indicaram que o livro também foi utilizado em brincadeiras, tornando-se uma forma de aprender se divertindo, fato potencial ao desenvolvimento infantil em meio à vivência do câncer^(10)^.

Estudo semelhante, conduzido no Japão, relatou o processo de criação de livro educativo para falar sobre câncer com crianças. Os resultados deste estudo japonês corroboram os achados do presente estudo, com profissionais e familiares indicando os benefícios do material para comunicação e acessibilidade da criança, indicando o aumento do seu conhecimento sobre a doença^(22)^. Todavia, esse livro se limitou a difundir informações apenas à criança, enquanto o “Guia Beaba do Câncer” pode ser utilizado pelas crianças, famílias e profissionais de saúde, avançando na fronteira do conhecimento.

Cabe refletir ainda que, historicamente, os serviços de saúde se organizaram em um padrão paternalista, em que a relação profissional e paciente cria empecilhos para comunicação aberta. Esse contexto leva crianças/adolescentes e familiares a se sentir pouco à vontade em fazer perguntas aos profissionais de saúde^(30)^. À luz do Interacionismo Simbólico, a ação pode ocorrer na dimensão “manifesta”, com a externalização de sensações, e na dimensão “encoberta”, com a experiência interna^(14)^. Fazendo uma associação dos dois aspectos citados, pode ser que, na prática clínica, a família não se sinta à vontade para fazer perguntas aos profissionais, mantendo a sua dúvida internamente, e os profissionais podem não dispender tempo para esclarecimento aprofundado de dúvidas. É digno de nota que o “Guia Beaba do Câncer” é um complemento às informações profissionais, não um substituto. O profissional pode utilizá-lo como instrumento facilitador de comunicação, mas deve dispender tempo adequado com as crianças/adolescentes e famílias^(11)^.

Para o futuro, indica-se a necessidade de estudos de implementação que possibilitem a aplicabilidade do “Guia Beaba do Câncer” em diferentes contextos da Rede de Atenção à Saúde, fazendo avaliações quanto ao seu uso para além do enfoque do paciente e familiar envolvido com o câncer, mas também para o público em geral, de forma a desmistificar estigmas sobre a doença. Ainda, apesar deste estudo ter incluído participantes de diferentes locais do Brasil, torna-se necessário analisar se adaptações semânticas serão necessárias para diferentes grupos culturais, visando um cuidado culturalmente responsivo.

### Limitações do estudo

Este estudo apresenta como limitações a prevalência de participantes residentes na região Sudeste do Brasil, o que pode ter restringido a diversidade de vivências culturais e regionais sobre a experiência investigada. A coleta de dados ter sido realizada exclusivamente por meio *online*, com a exigência de que os familiares fossem alfabetizados, o que pode ter excluído grupos sociais com menor acesso digital, letramento funcional limitado ou dificuldade de leitura do material educativo. Tais fatores impõem barreiras à inclusão de diferentes perspectivas, sobretudo de populações em maior vulnerabilidade social. O fato de o livro educativo ter sido entregue aos pacientes em um momento de fragilidade emocional pode ter influenciado positivamente suas percepções, gerando vínculos afetivos com o material que, embora legítimos, podem ter impactado as respostas dos participantes de forma subjetiva. Por fim, a escolha metodológica de ter incluído os participantes que já tinham familiaridade com o material educativo na etapa de validação anterior pode limitar a diversidade de percepções e restringir a generalização dos resultados, embora tenha assegurado maior profundidade na análise e continuidade no processo investigativo.

### Contribuição para a área da enfermagem

Este estudo contribui para a área da enfermagem, ao demonstrar a importância da integração de recursos lúdicos e educativos no cuidado assistencial, sendo o material “Guia Beabá do Câncer” uma estratégia potencial para transmissão de informações apropriadas à faixa etária de forma clara, acessível e acolhedora. Esse recurso pode ser utilizado na interação entre a criança, a família e os profissionais de saúde. Ademais, o material se mostra promissor para a prática de enfermagem, por dialogar com três pilares fundamentais da enfermagem pediátrica: (1) O direito das crianças e dos adolescentes, especialmente o direito de receber informações; (2) O respeito às necessidades essenciais da infância, reconhecendo suas experiências singulares; e (3) O cuidado centrado na família, ao promover o empoderamento dos familiares no processo de cuidar. Por fim, este estudo evidencia o potencial do livro como uma tecnologia leve de cuidado, fortalecendo a comunicação entre enfermeiros, crianças, adolescentes e seus familiares na prática clínica.

## CONSIDERAÇÕES FINAIS

Na perspectiva de crianças, adolescentes, familiares e profissionais de saúde, o “Guia Beaba do Câncer” apresenta uma linguagem lúdica e acessível, favorecendo a difusão do conhecimento e a desmistificação de informações desafiadoras sobre o câncer. Observou-se uma redução das reações negativas, além da promoção do empoderamento dos participantes e do fortalecimento das conexões interpessoais no contexto do câncer infantojuvenil.

## Data Availability

Os dados de pesquisa só estão disponíveis mediante solicitação.
